# End of the Season Blues: Senescence and Reproductive Trade-Offs in Male Scorpions

**DOI:** 10.3390/insects15120916

**Published:** 2024-11-23

**Authors:** David E. Vrech, Mariela A. Oviedo-Diego, Paola A. Olivero, Alfredo V. Peretti

**Affiliations:** Laboratorio de Biología Reproductiva y Evolución, Instituto de Diversidad y Ecología Animal (IDEA), CONICET-UNC, Facultad de Ciencias Exactas, Físicas y Naturales, Universidad Nacional de Córdoba Av. Vélez Sarsfield 299, Córdoba X5000, Argentina; dvrech@unc.edu.ar (D.E.V.); marie27oviedo@gmail.com (M.A.O.-D.); paoolivero@gmail.com (P.A.O.)

**Keywords:** reproduction, sexual selection, aging, sperm production, bothriuridae

## Abstract

Seasonal reproductive dynamics and senescence have profound impacts on male fertility, yet these processes remain understudied in scorpions. This study investigates how reproductive parameters—such as testicular mass, sperm quantity, and viability—change over the course of the reproductive season in *Urophonius achalensis* males. We found that early-season males exhibited higher sperm quality and testicular mass compared to their older counterparts, suggesting that senescence, rather than reproductive effort, drives the decline in sperm viability. Interestingly, isolated males accumulated more sperm without a reduction in viability, highlighting potential mechanisms that preserve sperm quality despite senescence. Body condition also played a key role, with males in better condition showing higher sperm counts but facing a sharper decline in sperm viability as the season progressed, suggesting trade-offs between early reproductive success and later-life reproductive costs. Our findings offer novel insights into the reproductive strategies of scorpions, emphasizing the interplay between senescence, environmental stressors, and reproductive investment. This work provides new insights into the reproductive biology of scorpions, with broader implications for understanding the role of senescence and mating systems in shaping reproductive strategies.

## Simple Summary

In this study, we explored how the reproductive biology of scorpion males changes over the reproductive season, particularly focusing on the quality and quantity of sperm. By comparing early- and late-season males, we found that testis size, sperm number, and sperm viability were higher at the beginning of the season but declined with age. Interestingly, even when males were isolated without mating opportunities, they still maintain sperm quality over time, suggesting potential preserving despite senescence. We also found that males in better physical condition produced more sperm but experienced a greater decline in sperm health as they aged, likely due to the energy demands of reproduction. These findings reveal the natural challenges male scorpions face as they age, highlighting the complex balance between reproduction and survival. Understanding these patterns is valuable as it provides insight into the broader issues of aging, reproductive health, and energy allocation in animals, which may help inform conservation efforts and may promote awareness about the biological effects of senescence in wildlife.

## 1. Introduction

Sexual and natural selection profoundly influence the often-divergent life-history traits between sexes [1,2,3]. Both males and females face different costs and decisions throughout their lives that shape their reproductive success [4,5]. Generally, males have shorter lifespans than females due to several factors, including engagement in riskier behaviors that lead to higher mortality rates, greater energetic investment in sexual traits, and differences in hormonal profiles and metabolic rates that contribute to accelerated aging [6,7,8]. In semelparous species [9], males often die at the end of the reproductive season as a consequence of the reproductive effort [10,11]. In contrast, females may exhibit longer lifespans, allowing them to give birth at the season’s end, with some individuals transitioning to the next reproductive season [12,13].

Each species has a defined mating period influenced by seasonality, which drives evolutionary adaptations that minimize the energy costs of reproduction in response to environmental changes [14,15,16]. Environmental factors like temperature, photoperiod, and food availability, as well as social factors such as female receptivity, affect mating timing and spermatogenesis [14,17,18]. During this period, both sexes undergo physiological and behavioral changes, with traits like testis size, sperm count, and viability fluctuating seasonally [19,20,21,22,23,24,25].

Sexual selection drives seasonal changes in male reproductive traits [26,27]. Sperm competition increases testes size and sperm production in some species [28,29,30,31,32,33], affecting seminal composition and sperm viability [34,35,36]. Sperm competition levels vary by season and population, and males adjust sperm production and allocation based on social cues [37,38]. Testes size and sperm storage peak during breeding seasons, while the male mating rate sequentially reduces ejaculate parameters like sperm count and spermatophore volume [39,40,41,42,43].

Seasonal changes in reproductive parameters may relate to aging, a time-dependent decline in biological function throughout life [44,45]. Senescence, a subset of aging, specifically refers to the gradual deterioration in health, reproductive capacity, and vitality after sexual maturity [46,47,48,49]. Senescence affects testes size and sperm traits, including viability and motility, with consequences for sperm reserves and competition [50,51,52,53,54,55]. Towards the end of the breeding season, testes may shrink or degenerate, reducing sperm production and altering sperm viability [56,57,58]. Ejaculate senescence occurs as older males have fewer resources [39]. Since sperm production is energetically costly, males often exhibit strategic sperm allocation across different mating events [37,59]. Sperm senescence is primarily studied in relation to both pre-meiotic and post-meiotic processes, affecting cells before and after meiosis, during sperm storage, and prior to ejaculation [60,61]. Damage during post-meiotic aging includes thermodynamic stress and oxidative damage (e.g., DNA damage by oxygen radicals) [62,63,64,65,66], increasing vulnerability to oxidative stress at different stages of sperm development.

Male age and mating history interact in complex ways, significantly affecting ejaculate quality [41]. In polygynous species, older males often have more mating experience, leading to higher cumulative reproductive effort and potentially more pronounced signs of senescence [67,68]. Understanding male reproductive senescence is crucial for predicting impacts on female reproductive fitness [69,70,71,72,73,74]. It is also important to determine whether sperm traits like quantity and quality decline together or if some are prioritized [75,76,77]. As sperm senescence likely arises in older males due to limited resources, given the high costs of sperm production [37,39], trade-offs between sperm traits may depend on body condition and resource availability, making senescence dynamics condition-dependent [65,68,78,79,80].

Seasonal changes and senescence effects on sperm have been widely studied in insects due to their short lifespans, diverse reproductive strategies, and pronounced changes in reproductive effort during their life cycles [81,82,83,84,85]. However, fewer studies have focused on arachnids, with some reports on spiders [86,87,88]. Arachnids are generally characterized by semelparous life cycles and polygynous males with continuous sperm production throughout their lifetime [87,89], but see [90,91]. Scorpions also produce sperm throughout the reproductive season. The scorpion reproductive system consists of two paraxial organs, producing hemispermatophores, connected to a two-lobed elastic seminal vesicle that stores sperm. One of those lobes connects to the testes tubes that produce the spermatozoa [92,93,94]. Regarding the dynamics of male sperm traits over time in scorpions, *Bothriurus bonariensis*, spermatophore regeneration rate slows towards the end of the season, and ejaculate volume decreases with successive matings [43,95]. Additionally, variability in sperm package morphology and intra-male sperm variability has been observed, with sperm number per package fluctuating over time [96,97,98]. However, there is a significant gap in understanding how male sperm parameters change over time in scorpions. Preliminary data suggest that sperm volume in seminal vesicles may vary with male age and season [99]. Additionally, testes may shrink or atrophy towards the end of the reproductive season.

The present study examines how seasonal changes impact male reproductive traits in a semelparous, polygynous scorpion species, *Urophonius achalensis* (Bothriuridae), native to high-altitude regions in central Argentina [100,101,102]. This species exhibits winter surface activity, facing harsh environmental conditions during its reproductive season [101,103,104]. We first examined the variation in reproductive variables of the scorpion *U. achalensis* males throughout the reproductive season (**Aim 1**). Due to the combined effects of male senescence, environmental conditions, and exposure to multiple matings during the reproductive lifespan, we hypothesize a deterioration in reproductive parameters, with a decline in both the quality and quantity of reproductive parameters (testes weight, seminal vesicle volume, sperm number, and viability) in males collected at the end of the season compared to those collected at the beginning. In addition, by isolating and preventing mating in a group of males collected at the beginning of the season but dissected towards the end, we directly analyzed the effect of sperm senescence by evaluating the variation in sperm cell number and viability (**Aim 2**). We anticipate a significant decline in the viability of these cells [105] in males that aged in isolation without mating, compared to males that aged in the field and were able to mate. This decline is likely due to the accumulation of sperm cells, aging (increased reactive oxygen species), and failures in the maintenance mechanisms for sperm, all of which would reduce sperm cell viability. Finally, we also expect a condition-dependent regime of reproductive parameters in males of different ages (**Aim 3**). Since the production and maintenance of reproductive structures, sperm, and accompanying substances can be physiologically costly activities [59,106], we predict that males in better condition will have heavier testes and higher sperm viability than those in poorer condition [43]. However, this condition-dependent regime may weaken or be lost in older males. For the number of spermatozoa or the volume of seminal vesicles, we expect a lower number in males of better condition collected towards the end of the season, reflecting a higher number of matings by these individuals.

## 2. Materials and Methods

### 2.1. Study Species, Collection, and Maintenance

It is important to note that it was not possible to rear individuals in the laboratory (maintenance and molting is difficult) [99], so the only way to obtain and analyze adults was to collect them directly in the field. Adult males of *U. achalensis* (n = 35) were collected throughout the reproductive season during the day by turning rocks from mid-April until the end of July 2019 from a population of the Sierras de Córdoba (31°22′21.3″ S, 64°46′03.9″ W; 1800 MASL), Argentina. As in other Bothriuridae [95,107], this species is semelparous, and males perish at the end of November [95,108]. Males at the beginning of the season are considered virgin because males do not survive longer than one reproductive season; therefore, old males from previous mating seasons cannot be found in nature [43]. Males of this species possibly present a polygynous mating system [108], unlike females that only mate once during their reproductive life (due to the presence of genital plugs) [109,110,111,112].

All males were kept in the same conditions and were dissected at different times to test the effect of seasonality and senescence on reproductive parameters. Individuals were maintained inside plastic containers (9 cm × 6 cm) with moistened cotton as a water supply and were fed with the larvae of *Tenebrio* sp. (Coleoptera, Tenebrionidae) every 7 days [110]. Food consumption was controlled and confirmed (i.e., every individual ingested the prey). All animals were kept under an inverted 11: 13 h. light: dark cycle and were maintained in temperatures of 0–5 °C during the dark phase and 10–15 °C during the light phase, with controlled humidity (65–80%). The temperature was modified according to records of the collection site to emulate the environmental conditions to which individuals are exposed in the wild. Voucher specimens have been deposited at the Laboratorio de Biología Reproductiva y Evolución (LaBRE), Facultad de Ciencias Exactas, Físicas y Naturales, Universidad Nacional de Córdoba, Argentina. The investigation adheres to the ASAB/ABS Guidelines for the Use of Animals in Research [113], and the use of animals was reviewed and approved by the animal care review committee at the Instituto de Diversidad y Ecología Animal (IDEA), where the experiments were carried out.

### 2.2. Experimental Groups

We classified males belonging to collection dates between mid-April and mid-May as “Early-season” (E) males (n = 12) and males collected from mid-May to the end of July as “Mid-to-late-season” (ML) males (n = 13). These males were collected directly from the field and were therefore exposed to natural environmental conditions and mating opportunities. For Aim 1, reproductive variables were compared between these groups of males.

On the other hand, for Aim 2, we compared the reproductive parameters of the “Mid-to-late-season” (field males) (MLF) with males that were collected earlier in the season but were kept isolated in the laboratory for 30 to 45 days to match the date of MLF collection (under controlled temperature and humidity conditions with no possibility of mating) (Early-season isolated) (I) (n = 10).

### 2.3. Dissections and Evaluation of Reproductive Parameters

Males were euthanized in a freezer at −20 °C for fifteen minutes and then dissected under a stereoscopic microscope (Nikon SMZ 1500, Nikon Corporation, Tokyo, Japan) in ‘spider saline solution’ (Saline solution: 3.26 g NaCl, 0.13 g KCl, 0.30 g CaCl_2_·2H_2_O, 0.26 g MgCl_2_·6H_2_O, and distilled water to a final volume of 250 mL) [114,115]. In the dissections, the male’s reproductive organs were extracted by making an incision in the mesosomal lateral pleura (between segments I and VII), exposing the paraxial organ. Digital images were taken using a phase-contrast microscope (Nikon Eclipse 50i, Nikon Corporation, Tokyo, Japan) with an attached digital camera (Nikon DS-fi1, Nikon Corporation, Tokyo, Japan). We considered different reproductive parameters: testicular quality and weight, total volume of seminal vesicles and potential ejaculate, number and viability of spermatozoa in the seminal vesicle.

#### 2.3.1. Testes Quality, Weight, and Seminal Vesicle Volume

**Figure 1:**
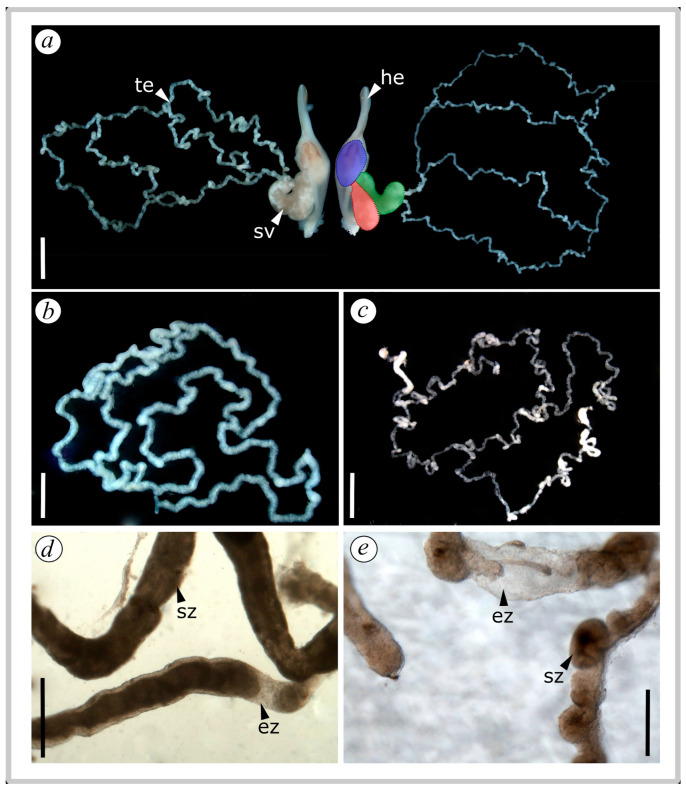
Reproductive system of *Urophonius achalensis* males dissected at different stages of the reproductive season, with details of the testes. (**a**) Hemispermatophores with seminal vesicles and testicles of males collected early in the season (left side), and towards mid-to-late season (right side). Note the difference in testicular turgidity. Color references: blue, potential ejaculate (sperm drop ready to be transferred to the female in next mating); red, store branch (blind branch of the seminal vesicle that stores sperm); green, connection branch (branch of the seminal vesicle connected to the testes). (**b**) Testes’ general structure of an early-season male. (**c**) Testes’ general structure of a male collected at mid-to-late season. (**d**) Details of spermatozoa in testes tubes and lumen of an early-season male. (**e**) Details of spermatozoa in testes tubes and lumen of a male collected at mid-to-late season. Note the presence of clearer and empty zones without sperm. Abbreviations: ez, empty-sperm zone; he, hemispermatophore; sv, seminal vesicle; sz, sperm zone; te, testes. Scale bars: a–c = 1 mm; d = 0.5 mm, e = 0.15 mm.

The testes of both paraxial organs were carefully extracted with fine tweezers and extended to take pictures of different sections. The turgidity of the testes was qualitatively analyzed by the presence of sperm filling the tube and its lumen. The occurrence of ‘empty’ spermatozoa zones along the structure was also recorded [99]. Subsequently, the testes were weighed to the nearest 0.0001 g on an electronic scale (Ohaus Pioneer PA114, Ohaus Corporation, Parsippany, NJ, USA) by triplicate (to account for measuring error) and the average of the measurements was used in the statistical analyses. We measured the total seminal vesicle volume from the digital images using the ImageJ measuring software package v. 1.54c [116] (Figure 1a). We estimated this volume from the ellipsoid equation (4/3 π a b c), where a, b, and c are the radiuses of the three axes measured from the images (following [43]). We also measured the potential ejaculate volume (serving as a proxy for sperm deposited inside the spermatophore and afterwards delivered to the female during insemination). considering the sperm drop formed at the base of the seminal vesicles (Figure 1a, blue section). This ejaculate volume was calculated using the same equation as the total seminal vesicle volume.

#### 2.3.2. Sperm Count and Viability

We cut the seminal vesicles longitudinally and removed their content with a 1–10 μL micropipette (Thermo Scientific Finnpipette, Thermo Fisher Scientific Inc, Waltham, MA, USA) (following [43]). The ejaculate was mixed inside a microcentrifuge tube with 50 μL of ‘spider saline solution’. To homogenize the sample, the solution was vortexed for 20 s using a shedding frequency of 40 Hz. Firstly, we analyzed spermatozoa viability placing a 10 μL sample on a glass slide with 1 μL of GelRed (Biotium Inc., Hayward, CA, USA) (dilution 1/1000) (following [117]). This DNA stain cannot penetrate living sperm cells [118] and dead cells emit red fluorescence when GelRed binds to their DNA. We calculated the proportion of live spermatozoa, counting 200 sperm cells in each sample (% sperm viability = [number of cells without staining/total number of cells counted] × 100) using an inverted epifluorescence microscope (Leica DiM8, Leica Microsystems, Wetzlar, Germany) (Filter cube RHODLP, excitation: 540/45, emission: 590). Following the estimation of viability, we counted sperm from the solution. In this case, the sample was vortexed for 2 min and adequately diluted, and spermatozoa were counted by duplicate in a Neubauer counting chamber, using a standard protocol [119].

### 2.4. Body Condition

Prior to dissection, each individual was weighed with an electronic scale (Ohaus Pioneer PA114). Following dissection, a digital picture of the individual’s prosoma (as a proxy of their total body size) was taken on a stereomicroscope with an ocular micrometer equipped with a digital camera [43,120]. Digital pictures were analyzed with ImageJ measuring software v. 1.54c [116]. Size and weight values were log-transformed and regressed to generate the residuals as an individual’s body condition index (residual index RI [121]).

### 2.5. Statistical Analysis

We analyzed the data with linear models (LM) and generalized linear models (GLM) in R 4.0.2 (http://www.r-project.org, accessed on 6 March 2023) [122]. Reproductive parameter traits were set as response variables: testes weight (g), total ejaculate volume in seminal vesicles (mm^3^), potential ejaculate volume (mm^3^), sperm viability (%), and spermatozoa number. In the case of the viability models, we constructed a response variable, with live cells as successes and dead cells as failures, to run a model with binomial error structure.

For Aim 1, these response variables were set, and the experimental group of males was placed as a fixed effect (“E”, “ML”) (see Section 2.2). In addition, we added the body condition in interaction with the experimental group (for Aim 3). For Aim 2, we set sperm number, vesicle volume, and sperm viability as response variables in different models. In this case, the fixed effect was the experimental group (“I”, “MLF”) (see Section 2.2), in interaction with body condition (for Aim 3).

Normality and homogeneity of variances were assessed graphically using the Cullen and Frey graph of the fitdistrplus package [123] and using the same package’s goodness-of-fit statistics. Data that did not follow normality were modeled according to the best distribution. Gamma (logit link function) and binomial (logit link function) distributions were modeled using the function ‘glm’ of the stats package provided by default on R installations [122], and negative binomial distributions (log link function) with ‘glm.nb’ function of the package MASS [124]. The validation of the fitted models was assessed graphically with residual analysis. We used the ‘visreg’ function and package to generate the graphs [125]. A significance level α of 0.05 was applied.

## 3. Results

### 3.1. Variation in Reproductive Parameters Traits Throughout the Season

**Figure 2:**
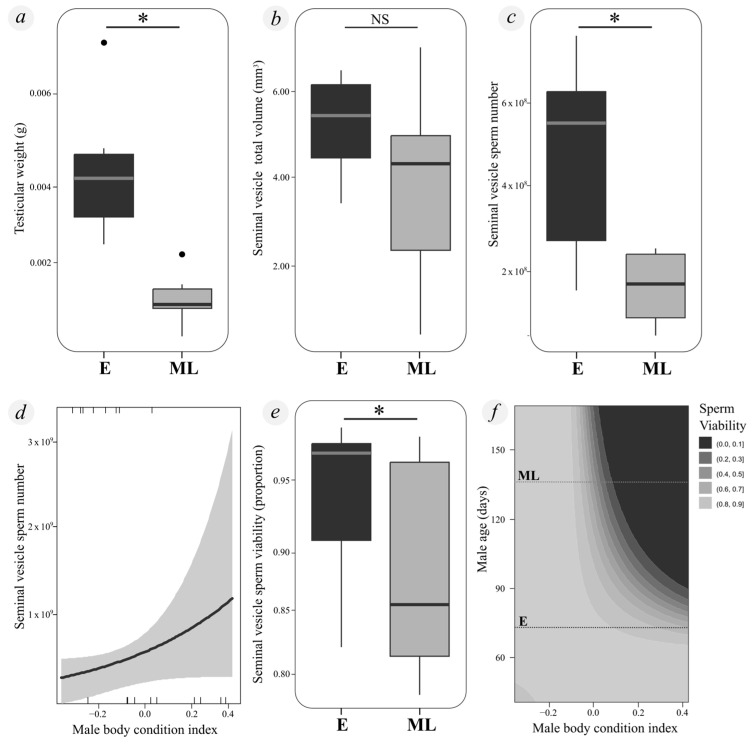
Differences in reproductive variables between experimental groups of *Urophonius achalensis* males throughout the season. (**a**) Testes weight (g) in male groups. (**b**) Effect of male’s experimental group on total seminal vesicle volume (mm^3^). (**c**) Spermatozoa number in different groups of males. (**d**) Relationship between body condition index and the number of sperm stored in the seminal vesicle, shaded gray area indicates the 95% confidence interval. (**e**) Proportion of sperm viability in different groups of males. (**f**) Contour plot showing the interaction between male age (considered as a continuous variable: the day on which the male was dissected as a continuous variable −0 days: 15 February, estimated date of last molt to adult) and body condition index on sperm viability. Darker shades of gray represent lower sperm viability, while lighter shades indicate higher viability. The labels “ML” and “E” mark specific age thresholds. The contour lines represent predicted probabilities of sperm viability, with values ranging from 0.0 to 0.9. References: *: Statistically significant difference between groups (*p*-value < 0.05); E: “Early-season” males collected between mid-April and mid-May; ML: “Mid-to-late-season” males collected from mid-May to the end of July; NS: Statistically significant differences not found. Box plots show 25% and 75% quartiles (boxes), medians (lines in the boxes), outermost values within the range of 1.5 times the respective quartiles (whiskers), and outliers (circles).

We found distinct qualitative differences in the turgor of the testes, with males collected early in the season (E) being much more turgid and firmer (with more spermatozoa collapsing the lumen and tubes) (Figure 1a,b,d), whereas mid-to-late-season males (ML) had a dilated appearance with many empty areas of spermatozoa (Figure 1a,c,e). Testicular weight was significantly higher in males collected early in the season (E) compared to those collected mid-to-late in the season (ML) (Chisq = 17.399, df = 1, *p* < 0.005) (Figure 2a). However, neither body condition (Chisq = 0.861, df = 1, *p* = 0.354) nor the interaction between experimental group and body condition had a statistically significant effect on testicular weight (Chisq = 0.124, df = 1, *p* = 0.724).

No significant differences were observed in the seminal vesicle total or potential ejaculate volume between E and ML males (seminal vesicle total volume: F = 1.359, df = 1, *p* = 0.274; potential ejaculate volume: F = 0.469, df = 1, *p* = 0.511) (Figure 2b). Additionally, these volumes were not significantly influenced by body condition (total volume: F = 0.071, df = 1, *p* = 0.796; potential ejaculate volume: F = 0.711, df = 1, *p* = 0.421) or by the interaction between body condition and experimental group (total volume: F = 0.006, df = 1, *p* = 0.938; potential ejaculate volume: F = 0.168, df = 1, *p* = 0.692).

Sperm count and viability were both significantly higher in E males compared to ML males (sperm count: Chisq = 15.661, df = 1, *p* < 0.005; viability: Chisq = 4.415, df = 1, *p* = 0.036) (Figure 2c,e). Males in better body condition also exhibited higher sperm counts (Chisq = 5.412, df = 1, *p* = 0.019) (Figure 2d), although there was no significant interaction between body condition and the experimental group for sperm count (Chisq = 0.209, df = 1, *p* = 0.648). However, a statistically significant interaction between experimental group and body condition was observed for sperm viability: while E males maintained consistently high viability across different body conditions, ML males in better body condition exhibited lower viability values (Chisq = 4.266, df = 1, *p* = 0.039) (Figure 2f).

### 3.2. Effect of Isolation on Sperm Quantity and Quality

**Figure 3:**
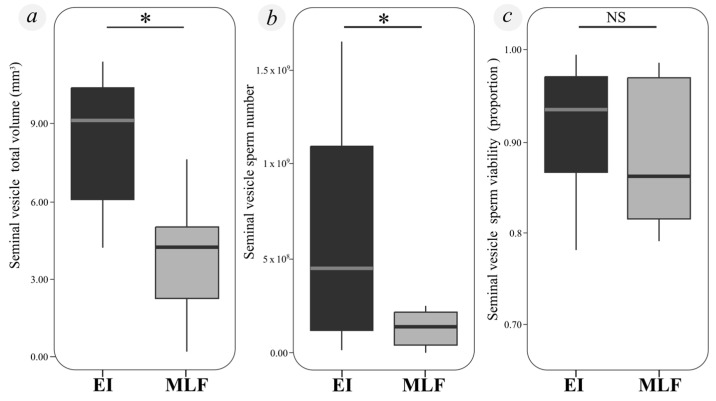
Differences in reproductive variable parameters between experimental groups of *Urophonius achalensis* males to examine sperm parameters in laboratory-isolated (I) and field-collected old males (MLF). (**a**) Effect of male’s experimental group on total seminal vesicle volume (mm^3^). (**b**) Spermatozoa number in different groups of males. (**c**) Proportion of sperm viability in different groups of males. Box plots show 25% and 75% quartiles (boxes), medians (lines in the boxes), outermost values within the range of 1.5 times the respective quartiles (whiskers). References: I: “Early-season” males collected between mid-April and mid-May and isolated until the end of the season; MLF: “Mid-to-late-season” field males collected from mid-May to the end of July; NS: Statistically significant differences not found; *: Statistically significant difference between groups (*p*-value < 0.05).

When comparing males collected at the beginning of the season but isolated (I) with those collected directly from the field mid-to-late in the season (MLF), we found that I males had significantly greater total vesicular volume and potential ejaculate volume (total volume: F = 12.089, df = 1, *p* = 0.003; transference volume: F = 8.177, df = 1, *p* = 0.012) (Figure 3a). These volumes were not significantly affected by body condition (total volume: F = 0.053, df = 1, *p* = 0.822; potential ejaculate volume: F = 1.035, df = 1, *p* = 0.325) or by the interaction between body condition and experimental group (total volume: F = 0.266, df = 1, *p* = 0.613; transference volume: F = 0.008, df = 1, *p* = 0.929).

Regarding sperm count, I males had significantly higher numbers of sperm in their vesicles compared to MLF males (Chisq = 4.621, df = 1, *p* = 0.032) (Figure 3b). Neither body condition (Chisq = 0.105, df = 1, *p* = 0.746) nor the interaction between body condition and experimental group (Chisq = 0.909, df = 1, *p* = 0.340) significantly affected sperm count. No significant differences in sperm viability were observed between experimental groups (Chisq = 0.209, df = 1, *p* = 0.647) (Figure 3c), and sperm viability was not significantly influenced by body condition (Chisq = 0.001, df = 1, *p* = 0.973) or by the interaction between body condition and experimental group (Chisq = 1.037, df = 1, *p* = 0.309).

## 4. Discussion

This study is the first to investigate seasonal variations in reproductive parameters using scorpions as a study organism. We identified significant differences in testes quality and sperm quantity throughout the reproductive season, notably influenced by senescence. The absence of variations in ejaculate volume raises questions about the role of accessory substances affecting these patterns. Our analysis revealed that variables such as sperm viability also decline over the course of the season. In the male isolation experiment, we observed an abrupt increase in sperm count but no decrease in viability in isolated males relative to wild-caught males. This pattern suggests that senescence, rather than reproductive activity, plays a dominant role in the decline in sperm viability observed in old males. Although our predictions regarding a condition-dependent regime with certain spermatic traits were not fulfilled, we obtained interesting interactions between male condition and age on sperm viability. These findings illustrate the natural variations undergone by males and their possible associated trade-offs, underscoring the challenges faced by adult male scorpions during the reproductive season.

### 4.1. Seasonal Changes in Sperm Production and Storage in U. achalensis Males

As predicted, males collected and analyzed at the beginning of the reproductive season exhibited heavier testes, a greater number of spermatozoa, and higher sperm viability compared to those collected later in the season. These parameters declined both in quantity and quality as the season progressed. For example, in addition to decreasing in weight, the testicles of older males showed loss of turgidity and a higher number of empty-sperm zones, which could be affecting the reproductive capacity of these males at some level [126]. This gradual deterioration is undoubtedly multicausal and, although the precise discernment of the factors with the greatest impact on the decline of these parameters is beyond the scope of this paper, we can speculate on the joint effects of aging, environmental conditions and mating history on males.

Similar age-related declines in reproductive parameters have been observed in vertebrates, where aging is associated with increased collagen fiber volume in the testes and reduced testosterone synthesis [127,128]. Studies indicate that a high mating rate can directly influence testicular characteristics [40,129]. In insects, males deprived of mating opportunities do not show decreased testes mass [130]. The intense activity of spermatogenic tissue during the mating season may accelerate aging processes, suggesting that testes mass in *U. achalensis* males could be influenced by various factors, including mating rate and sperm allocation. However, unlike other highly polygynous species [43,95,120], preliminary observations suggest that the rate of sexual encounters and matings is not so high in this species [99]. It should be noted that the main reproductive period lasts about three months, with a regeneration of spermatophores every 10–15 days [95], and that no more than 6–7 matings could be expected during the life of each male (assuming that the individual survives the winter and is not cannibalized or predated beforehand). We consider that other factors are possibly more important in the degradation of reproductive parameters in this species, in particular the senescence of the males as well as the effect of harsh environmental conditions during the season.

Testicular atrophy corresponds with reduced sperm numbers in older males collected later in the season. While mating history could account for this decrease, it is unclear whether there is also a process of sperm reabsorption or phagocytosis as happens in other groups of vertebrates and invertebrates (e.g., [131,132,133]). Currently, data on scorpion sperm reabsorption or phagocytosis presence and rates are lacking. Additionally, sperm viability declines throughout the season, with early-season males exhibiting high sperm viability compared to older males collected later in the season. This decline could be attributed to both aging and the reduced reproductive opportunities as the season progresses. The unique mating system of *U. achalensis*, where the females have a mating plug and the operational sex ratio is male-biased due to females being removed from the mating pool after insemination [108,111,112], further accentuates a drop in mating opportunities towards the end of the season. This characteristic may or may not have consequences for female fitness: (a) if all females are mated and plugged early in the season, a drop in viability would have no consequences; however, if (b) some females remain virgins and mate towards the end of the season, they would receive aged sperm (which would interact with female age).

The technique applied to measure sperm viability had previously been used in spiders [117,134,135] but had not been successfully used in scorpions until now. We observed high sperm viability in males, with more viable spermatozoa in younger males. This high sperm viability has also been reported in spiders [117] and may be partly attributed to sperm being encapsulated by a protein sheath surrounding the spermatozoa, which may play a protective role [136]. While scorpions do not possess such a capsule, most families have spermatozoa gathered in sperm packages [94,96]. Although it has been speculated that these sperm packages may be linked to increased sperm viability, this hypothesis has not yet been tested [97]. Interestingly, Vrech et al. [98] reported that sperm package morphology might vary with age in the scorpion *Brachistosternus ferrugineus*. Thus, future studies linking sperm package morphology to viability could help elucidate the mechanisms underlying the observed patterns.

In contrast, we did not observe a decrease in seminal vesicle volume towards the end of the season. It is important to clarify that the ejaculate is not composed solely of sperm, but also contains accessory substances [106,137,138]. Although the spermatozoa number declines throughout the reproductive season, the accessory substances may not follow the same pattern, which could explain why the volume of the seminal vesicle does not decrease. Some studies have shown that, while the quantity of accessory substances may remain stable, their quality can decline over time [85,139]. This deterioration could affect sperm parameters such as viability or motility and may even have consequences for the fitness of females and offspring [140]. In a closely related species, *U. brachycentrus*, granular accessory substances have been found in both the seminal vesicles and spermatophores, which were later detected in females [99]. However, little is known about this phenomenon in scorpions, and even less is known about the chemical composition and potential temporal variation in the quality of the ejaculate accessory substances.

Similarly, just as we did not observe seasonal changes in the total ejaculate volume, we also did not find such changes in the potential ejaculate volume. This partially contrasts with the findings of Vrech et al. [43] in *B. bonariensis*, where a 13% decline in ejaculate volume was observed in spermatophores over a period of approximately one month. However, it is important to consider that in that study, males were subjected to intensive re-mating, and the ejaculate volume was measured directly from the spermatophores. Additionally, the method we used in this work to estimate the “potential ejaculate” from the seminal vesicle may be innovative, but it may not accurately reflect what happens in the spermatophore (e.g., the addition of other accessory substances, sperm allocation). Future studies will need to investigate variation in ejaculate quantity directly from the spermatophore, rather than the potential volume from the seminal vesicle [126,141].

### 4.2. Sperm Senescence in Wild and Isolated Old Males

Our experimental design allowed us to compare reproductive parameters between old males that remained in the field under natural conditions (MLF) and those isolated in the lab without mating opportunities (I). As expected, isolated males accumulated more than double the sperm and had a higher ejaculate volume due to their inability to mate, compared to MLF. A similar pattern was observed in male mosquitofish (*Gambusia holbrooki*) where sexually active males showed fewer sperm and had a lower rate of sperm replenishment than males prevented from mating [41]. Both groups of older males exhibited lower sperm viability than younger males, but contrary to our predictions, there were no significant differences in viability between the two older groups. This suggests that the decline in sperm viability towards the end of the season is primarily due to senescence rather than repeated matings or environmental factors.

Prolonged sperm accumulation in seminal receptacles can lead to an excessive amount of reactive oxygen species (ROS) (a by-product of aerobic metabolism and environmental stress), potentially resulting in reduced sperm quality [54,60,142,143]. However, this quality drop was not observed in isolated males (I), where a high accumulation of sperm was detected. This result is particularly intriguing, and although the underlying causes of this phenomenon are unknown, several non-mutually exclusive explanations could be proposed: (a) the specific action of seminal fluid or accessory substances that may preserve the sperm, (b) high resilience in sperm viability due to the presence of sperm packages [97], and (c) a terminal strategy in males that have not mated, maintaining high sperm viability. Much remains to be understood regarding the ejaculate physiology and the potential reproductive strategies in scorpions, but disentangling the factors contributing to variations in ejaculate quality is a first step in this understudied group.

### 4.3. Relationship Between Body Condition and Reproductive Variables

We found partial support for our initial hypothesis regarding the relationship between body condition and reproductive traits. On the one hand, we did not obtain support for a condition-dependent regime for testicular weight and total and potential ejaculate volume. Besides, males in better condition had more spermatozoa in their vesicles, consistent with other invertebrate studies [144]. A previous study on *B. bonariensis* found that ejaculate volume was positively related to male size but not to sperm number [43]. While this study focused on a different species and quality indicator (body condition), the discrepancies could be attributed to the different mating systems of these species [111,112,120]. In *U. achalensis* females could be much more selective during the precopulatory period than those of *B. bonariensis* because they are monandrous, and we know that body condition is a trait assessed by females [102]. In this case, it could be that male body condition is an honest indicator of male quality for sperm competition (higher sperm number).

We found no support for our prediction of a lower number of spermatozoa in older males of better condition due to increased usage in mating. Notably, we did observe the expected pattern for sperm viability that was influenced by the interaction of male condition and age. In younger males, body condition did not affect viability, with high sperm viability across all different conditions. However, as the reproductive season progressed, males in better condition were more severely compromised in viability than males in poor body condition. As previously stated, females are more likely to mate with males in better condition [102], so these males at the end of the season may have had increased sexual activity, which can be reflected in a decrease in the quality of the remaining sperm. This relationship may reflect trade-offs between early- and late-life reproductive performance [145], with initially successful males experiencing greater reproductive costs later in the season. However, this accelerated senescence experienced by better-conditioned males could be attributed to multiple causes beyond sexual activities, such as increased energy demands, greater exposure to predators, and heightened indirect competition with other males.

## 5. Conclusions

Our study provides the first comprehensive examination of seasonal variations in reproductive parameters in scorpions, with a focus on *Urophonius achalensis* males. Our findings highlight the significant influence of senescence on sperm quality and reproductive capacity, revealing a gradual decline in testicular mass, sperm quantity, and viability over the course of the reproductive season. Notably, while ejaculate volume remained stable, the observed decrease in sperm quality suggests potential trade-offs between reproductive investment and other life history traits as the mating season progresses.

The present work also sheds light on the role of isolation and reproductive activity in sperm viability. Isolated males showed a marked increase in sperm accumulation without a corresponding decline in viability, emphasizing the dominant effect of senescence over reproductive effort in determining sperm quality. This result invites further research into the physiological and biochemical mechanisms that may preserve sperm in scorpions, such as the role of sperm packages or accessory substances.

The relationship between male body condition and reproductive traits proved complex. Better-conditioned males showed higher sperm numbers but experienced a more pronounced decline in sperm viability later in the season. This suggests a trade-off between early reproductive success and later-life reproductive costs, potentially driven by increased mating activity in high-condition males.

Overall, our findings provide valuable insights into the reproductive strategies of *U. achalensis* males and the challenges they face throughout the reproductive season. By identifying the key factors influencing sperm production, storage, and viability, this study lays the groundwork for future investigations into the reproductive physiology of scorpions, particularly in relation to the effects of senescence and environmental stressors. Further studies on ejaculate composition and the adaptive significance of sperm packaging are needed to fully understand the reproductive trade-offs and strategies employed by male scorpions in natural settings.

## Data Availability

Dataset available on request from the authors. The raw data supporting the conclusions of this article will be made available by the authors on request.
